# Enteroinvasive *Escherichia coli* May Account for Uncultured *Shigella*

**DOI:** 10.4269/ajtmh.15-0777a

**Published:** 2016-02-03

**Authors:** Iruka N. Okeke, Aaron O. Aboderin, Japheth A. Opintan

**Affiliations:** Faculty of Pharmacy, University of Ibadan, Ibadan, Nigeria E-mail: iruka.n.okeke@gmail.com; Department of Medical Microbiology and Parasitology, College of Health Sciences, Obafemi Awolowo University, Ile-Ife, Nigeria. E-mail: diipo_aboderin@yahoo.com; Department of Medical Microbiology, College of Health Sciences, University of Ghana, Accra, Ghana. E-mail: jaopintan@ug.edu.gh

Dear Sir:

A recent article by Lindsay and others^1^ reported that stool quantitative polymerase chain reaction (qPCR), with a 14,000 copy number cutoff, identified more cases of *Shigella* infection than conventional and widely used culture methods. The authors suggested that there may be a significant underestimation of the contribution of *Shigella* to diarrheal disease because of the limits of culture and have made a similar claim in a previous but smaller study.^2^ The target gene for *Shigella* used in the study was *ipaH*, one of the most robust and widely used *Shigella* targets. As some of the authors remarked in a previous article, enteroinvasive *Escherichia coli* (EIEC) strains also possess *ipaH*.^2^ Indeed, this target is applied for both *Shigella* and EIEC when the two pathotypes are not delineated. It is therefore possible, or indeed probable, that the qPCR methodology has uncovered a hidden burden due to EIEC rather than—or with—missed *Shigella* cases.

EIEC strains are less commonly sought overall than *Shigella* since they cannot be reliably delineated biochemically from commensal *E*. *coli* by biochemical and serological testing alone.^3^ They are genetically more heterogeneous than *Shigella* and only detectable by invasion assays and molecular tests.^4^ The Global Enteric Multicenter Study from which these findings emanate sought a wider range of pathogens than most, including some diarrheagenic *E*. *coli*, but not EIEC.^5^ Therefore, the only presumptive data pointing to EIEC from that study are the *ipaH* qPCR data. We note with particular interest from the report of Lindsay and others^1^ that the proportion of “shigellae” that were uncultured, as determined from qPCR data, varied by site. There might be technical, stability, host biology, or coinfection explanations for this finding. However, a more plausible explanation is that EIEC are more prevalent at the African sites where *Shigella* culture was less sensitive than in Bangladesh where most “*Shigella*” infections were culture positive. This presumption is backed up by the finding by Lindsay and others^1^ that the distribution of *Shigella* serotypes varied among the sites. The greater diversity of EIEC lineages may additionally explain why culture-negative *ipaH*-positive cases were more common in older children.

*Shigella* and EIEC are both *E*. *coli* pathotypes, both are known to produce dysentery (and therefore require antimicrobial chemotherapy) and to have low infection doses. In spite of these similarities in virulence, and the close relationships between some EIEC and *Shigella* lineages,^6^ harder-to-detect EIEC are often overlooked in epidemiological studies and routine diagnosis are presumed to be less significant in the absence of a robust evidence base.^2^,^3^ If EIEC accounts for some or all of the excess detection produced by *ipaH* qPCR, the epidemiological data reported in the article by Lindsay and others^1^ suggest that it is as important to detect EIEC as *Shigella* since nutritional status and risk factors were similar in culture and *ipaH* qPCR-positive cases. Perhaps the most important and completely unstated finding of the study is that alternatives to culture are essential for capturing the burden from all *ipaH*-positive pathogens, and more importantly delineating EIEC from *Shigella*. It is critical to highlight that the findings of the Lindsay and others^1^ study point to epidemiological importance of EIEC, particularly at African sites, to properly inform future studies and vaccine development.
